# Proportion of Pelvic Inflammatory Disease Cases Caused by *Chlamydia trachomatis*: Consistent Picture From Different Methods

**DOI:** 10.1093/infdis/jiw178

**Published:** 2016-06-03

**Authors:** Malcolm J. Price, A. E. Ades, Nicky J. Welton, Ian Simms, John Macleod, Paddy J. Horner

**Affiliations:** 1 Institute of Applied Health Research, University of Birmingham,; 2 School of Social and Community Medicine, University of Bristol; 3 Public Health England, London; 4 Bristol Sexual Health Centre, University Hospital Bristol NHS Foundation Trust, United Kingdom

**Keywords:** *Chlamydia trachomatis*, pelvic inflammatory disease, population attributable fraction, population excess fraction, meta-analysis, Bayesian, evidence synthesis

## Abstract

***Background.*** Pelvic inflammatory disease (PID) is a leading cause of both tubal factor infertility and ectopic pregnancy. *Chlamydia trachomatis* is an important risk factor for PID, but the proportion of PID cases caused by *C. trachomatis* is unclear. Estimates of this are required to evaluate control measures.

***Methods.*** We consider 5 separate methods of estimating age-group-specific population excess fractions (PEFs) of PID due to *C. trachomatis*, using routine data, surveys, case-control studies, and randomized controlled trials, and apply these to data from the United Kingdom before introduction of the National Chlamydia Screening Programme.

***Results.*** As they are informed by randomized comparisons and national exposure and outcome estimates, our preferred estimates of the proportion of PID cases caused by *C. trachomatis* are 35% (95% credible interval [CrI], 11%–69%) in women aged 16–24 years and 20% (95% CrI, 6%–38%) in women aged 16–44 years in the United Kingdom. There is a fair degree of consistency between adjusted estimates of PEF, but all have wide 95% CrIs. The PEF decreases from 53.5% (95% CrI, 15.6%–100%) in women aged 16–19 years to 11.5% (95% CrI, 3.0%–25.7%) in women aged 35–44 years.

***Conclusions.*** The PEFs of PID due to *C. trachomatis* decline steeply with age by a factor of around 5-fold between younger and older women. Further studies of the etiology of PID in different age groups are required.

Pelvic inflammatory disease (PID) is a leading cause of both tubal factor infertility (TFI) and ectopic pregnancy [1, 2]. PID is a clinical diagnosis typically indicated by lower abdominal pain with local tenderness on bimanual examination [3]. There are around 50–75 000 PID cases diagnosed in England annually, around one third of which are in women aged 16–24 years [4]. An unknown proportion—perhaps as much as 70%—of incident PID cases remain undiagnosed [3, 5]. *Chlamydia trachomatis* is an important risk factor for the development of PID. But PID is also caused by other infections of the female reproductive tract [6]. With the many different causes of PID, the extent of the role of *C. trachomatis* in the etiology of PID is unclear.

While the importance of *C. trachomatis* in the etiology of PID is widely accepted, attempts to quantify this are limited. Based on 19 studies reporting *C. trachomatis* prevalence in women with PID from 1977 to 1992, Paavonen et al [7] reported that *C. trachomatis* was involved in 30% of PID cases. Simms and Stephenson [8] summarized studies of *C. trachomatis* prevalence in women with laparoscopically proven PID. The proportion with evidence of current *C. trachomatis* in upper genital tract samples varied from 12% to 61%, reflecting large variation over time and place. However, these studies lacked a control group, and results depended markedly on the sites from which samples were taken.

In this article, we describe 5 separate although not completely independent methods to estimate the population excess fraction (PEF) of PID due to *C. trachomatis*. The PEF is the most commonly used form of population attributable risk fraction and is defined as the proportional reduction in disease risk that would be achieved by eliminating the exposure of interest from the population, assuming the exposure is causally related to the disease [9]. PEF is a property not only of the disease and the exposure, but also of the time and place where the data were collected. When estimating the PEF for a given setting, there are a variety of data sources with which a credible estimate should be consistent. For example, the ratio of the incidence of PID to the incidence of *C. trachomatis* infection must be the same as the ratio of the risk of PID from *C. trachomatis* to the proportion of PID episodes caused by *C. trachomatis*. As such, estimates of any 3 of these quantities can be used to calculate the fourth, and for independent estimates of all 4 to be coherent they must be consistent subject to this constraint.

We apply the methods to United Kingdom data to develop estimates for the period immediately before the onset of the National Chlamydia Screening Programme, in 2003 [10]. It has generally been overlooked that, while both *C. trachomatis* infection and PID share the same downward trend in incidence with age, the decline for *C. trachomatis* infection is far steeper and occurs at younger ages [4]. There are several plausible explanations for this. The proportion of PID cases diagnosed may increase with age, the risk that *C. trachomatis* causes PID may be higher in older women, or the proportion of PID episodes caused by *C. trachomatis* may decline with age [4]. Therefore, age is an important covariate to consider, especially as it is used to define the target population for *C. trachomatis* screening.

We consider each of the 5 methods in turn. In each case, we review the literature to identify relevant data sources to estimate the model parameters, performing evidence synthesis where appropriate. We describe the mathematical relationship between the PEF and the parameters that the data sources estimate. A Bayesian approach is adopted to ensure correct propagation of uncertainty from all evidence sources. Finally, we review the various estimates of PEF and consider the strengths and weaknesses of each method.

## METHODS

We briefly describe the 5 methods for estimating the PEF, in turn. Each method shows how a certain type of data can be used. In each case, we set out the relevant data sources for the United Kingdom and any statistical models used to analyze them, and we derive the joint functional relationships between the parameters they provide estimates of and the PEF.

### PEF Estimate 1 (PEF-1): Crude Estimate From Case-Control Studies and Age-Specific *C. trachomatis* Infection Prevalence Estimates

Case-control studies are commonly used to estimate PEFs. The standard formula for estimating the PEF from case-control data [9] is

$$\text{PEF = }\frac{\pi .(\mathrm{OR}-1)}{\pi .(\mathrm{OR}-1)+1},$$

where OR is the odds ratio (assumed to approximate the incidence rate ratio) of the disease in the exposed group relative to that in the unexposed groups and *π* is the prevalence of the exposure in the population of interest. The formula is only correct when there are no confounding factors or when all confounders have been adjusted for in the estimation of the odds ratio. The impact of confounding in observational studies of *C. trachomatis* and PID is likely to be significant as the risk factors for *C. trachomatis* infection are similar to the risk factors for many other causes of PID (eg, other sexually transmitted infections [STIs]).

Numerous case-control studies comparing measures of *C. trachomatis* exposure in women with and those without PID have been published. We only consider case-control studies that use current infection as a marker of exposure as the risk of confounding is lower than in studies using measures of cumulative exposure, such as serological data [4]. Furthermore, we only consider European studies, because the epidemiology of STIs is generally similar in Western European countries, and we exclude those published before the 1990s.

Our search identified 3 studies (Table 1) [11–13], none of which stratified by age group or reported sufficient data to attempt to adjust for confounders. The studies were pooled using a fixed-effect logistic regression model to obtain a pooled estimate of the OR. Further details of the search strategy and statistical methods are provided in Appendix 1.

**Table 1.** Data and Crude Odds Ratios (ORs) From Retrospective Studies Used in Population Excess Fraction Estimates 1 and 2

Study, Group	Data^a^	Crude *C. trachomatis* Prevalence (95% CI)	Crude OR (95% CI)
Paavonen et al [12]			
Cases	13/30	0.43 (.28–.63)	6.9 (.8–61.4)
Controls^b^	1/10	0.10 (.03–.44)	
Mascellino et al [11]			
Cases	22/110	0.20 (.14–.29)	7.0 (3.1–15.8)
Controls	9/261	0.03 (.02–.06)	
Simms et al [13]			
Cases	17/140	0.12 (.08–.19)	18.7 (2.5–142.1)
Controls^b,c^	1/136	0.01 (.00–.04)	
Pooled estimate	…	…	9.2 (4.4–18.1)
Pooled adjusted	…	…	17.1 (7.9–34.0)

Abbreviations: CI, confidence interval; *C. trachomatis*, *Chlamydia trachomatis*.

^a^ Binomial numerators and denominators.

^b^ Confidence intervals are illustrative because there are insufficient numbers to assume asymptotic normality.

^c^ GP control group used.

Estimates of the prevalence of *C. trachomatis* infection in women in England by age group are available from a recent synthesis of evidence from prevalence, incidence, and duration studies [14] (Table 2). The first PEF estimate is calculated as follows:

$${\text{PEF}}_{a}^{\left(1\right)}=\frac{\pi_{a}.({\mathrm{OR}}_{a}-1)}{\pi_{a}.({\mathrm{OR}}_{a}-1)+1}.$$

**Table 2.** Estimates of the Prevalence of *Chlamydia trachomatis* Infection Among Women Who Ever Had a Sex Partner

Age	*C. trachomatis* Prevalence, %, Mean (95% CrI)
16–19 y	6.7 (4.5–9.5)
20–24 y	4.0 (2.9–5.3)
25–34 y	1.2 (0.8–1.6)
35–44 y	0.8 (0.5–1.3)
16–24 y	5.2 (3.8–6.9)
16–44 y	2.1 (1.6–2.7)

Data were obtained from a synthesis of data on *C. trachomatis* infection duration, incidence, and prevalence from England, as reported by Price et al [14], and used in population excess fraction (PEF) estimates 1–4.

Abbreviation: CrI, credible interval.

Age groups 16–19, 20–24, 25–34, and 35–44 years are indexed by *a*. Owing to case-control studies reporting insufficient information, the formula assumes that the OR is constant across age strata (see “Discussion” section).

### PEF-2: Estimate From Case-Control Studies Adjusted for Ascertainment Error and Age-Specific Prevalence of *C. trachomatis* Infection

Estimates of the prevalence of *C. trachomatis* infection in women with PID differ markedly depending on the sites from which samples are collected. The 3 studies [11–13] identified above used samples from the lower genital tract. However, the presence of *C. trachomatis* in the upper genital tract, which is more likely to be causally related to PID, may not be well predicted by its presence in the lower genital tract [15, 16]. A recent study in Erfurt, Germany [16], looked at 363 women with laparoscopically confirmed PID. *C. trachomatis* was found in the genital tract of 103 (28.4%), and in 55 (15.2%) it could be isolated from the cervix. In 23 (6.3%), *C. trachomatis* was isolated from both the cervix and the fallopian tubes, while in 47 (12.9%), *C. trachomatis* was isolated from the fallopian tubes only. We use data from the study by Erfurt et al to attempt to adjust for underascertainment of *C. trachomatis* infection among the PID cases:

$${\text{PEF}}_{a}^{\left(2\right)}=\frac{\pi_{a}.((\mathrm{OR}/\psi )-1)}{\pi_{a}.((\mathrm{OR}/\psi )-1)+1},$$

where *ψ* is the proportion of all cases of *C. trachomatis* infection in women with PID in which the organism is isolated from the lower genital tract. Details of how data from the study by Erfurt et al are used to estimate *ψ* are given in Appendix 1.

### PEF-3: Crude Estimate From Screening Trials and Age-Specific Estimates of the Prevalence of *C. trachomatis* Infection

The PEF can be estimated using prospective studies. Observational studies will also provide estimates that are vulnerable to significant confounding. We therefore consider estimates from randomized controlled trials (RCTs). Several RCTs designed to estimate the effect of different types of screening have been performed [17–19]. In 2, only the screened arm was tested at baseline. However, in the POPI trial [17], one arm was screened and treated immediately, while in the other arm samples were collected but not tested until follow-up, 12 months later. Thus, the relative risk (RR) of PID in women known to be *C. trachomatis* positive at baseline (7 of 74 developed PID), compared with women who are *C. trachomatis* positive but screened and treated and who are assumed to be *C. trachomatis* negative at baseline (1 of 63 developed PID), can be estimated. Hence, the PEF can be calculated using an estimate of the RR, based on a randomized comparison:

$${\text{PEF}}_{a}^{\left(3\right)}=\frac{\pi_{a}(\mathrm{RR}-1)}{\pi_{a}(\mathrm{RR}-1)+1}.$$

The RR is the relative risk of developing PID in the exposed versus unexposed group and approximates the incidence rate ratio. The trial was performed in younger women (range, 16–27 years; 89% were aged ≤24 years). Note that estimates for age groups 25–34 years and 35–44 years should be treated with caution as they extrapolate beyond the age range of the POPI trial.

### PEF-4: Crude Estimate From Screening Trials Adjusted for Treatment and Age-Specific Prevalence of *C. trachomatis* Infection

The POPI protocol advised all women in the deferred screening arm to be independently tested. Of the *C. trachomatis*–positive women in this arm, 43% were tested and treated during follow-up. We assume that testing occurred randomly during follow-up so that each of the 32 patients (74 × 43% = 32) who were treated are at risk from the initial infection for a random time between 0 and 1 years. Therefore, the adjusted proportion of cases is calculated as

$$\begin{array}{cc} & \omega =\frac{42+\sum_{i=1}^{32}\;X_{i}}{74}\\ & X_{i}∼\text{Uniform(0,1),} \end{array}$$

where ${\text{PEF}}_{a}^{\left(3\right)}$ can thus be adjusted to allow for the effect of treatment during follow-up, as follows:

$${\text{PEF}}_{a}^{\left(4\right)}=\frac{\pi_{a}((\mathrm{RR}/\omega )-1)}{\pi_{a}((\mathrm{RR}/\omega )-1)+1}.$$

### PEF-5: Estimate-Based Synthesis of Screening Trials and Age-Specific *C. trachomatis* Infection and PID Incidence

A final method to estimate the PEF is the ratio of the incidence of PID caused by *C. trachomatis* to the incidence of all-cause PID, calculate as

$${\mathrm{PEF}}_{a}^{\left(5\right)}=\frac{I_{a}^{\mathrm{CT}}R^{\mathrm{CT}->\mathrm{PID}}}{I_{a}^{\mathrm{ALL}\;\mathrm{PID}}},$$

where $I_{a}^{\mathrm{CT}}$ is the incidence of *C. trachomatis*, $I_{a}^{\mathrm{ALL}\;\mathrm{PID}}$ is the incidence of all-cause PID in England, and $R^{\mathrm{CT}->\mathrm{PID}}$ is the risk of PID from a single *C. trachomatis* episode until the point of treatment or clearance.

Estimates of *C. trachomatis* incidence are available from the synthesis of incidence, prevalence, and duration studies previously cited (Table 3) [14]. For the risk that *C. trachomatis* causes PID (excess risk), we use an estimate of 17.1% (95% credible interval [CrI], 5.6%–28.9%) taken from a recent synthesis of data from 3 RCTs of screening interventions [20].

**Table 3.** Estimated Incidence Rates of *Chlamydia trachomatis* Infection and Pelvic Inflammatory Disease (PID) Among Women in England

Age	Incidence, Cases/100 Person-Years, Mean (95% CrI)	
	*C. trachomatis* Infection	PID
16–19 y	6.5 (4.2–9.6)	2.1 (1.5–2.9)
20–24 y	3.9 (2.7–5.4)	2.8 (2.0–3.9)
25–34 y	1.1 (0.7–1.7)	1.9 (1.3–2.8)
35–44 y	0.8 (0.5–1.3)	1.3 (0.8–1.9)
16–24 y	5.0 (3.5–7.0)	2.5 (1.8–3.4)
16–44 y	2.1 (1.5–2.8)	1.8 (1.3–2.5)

Data on *C. trachomatis* infection are from Price et al [14], and data on PID are from Price et al [4].

Abbreviation: CrI, credible interval.

There are 3 sources of routine data on PID incidence in England: Hospital Episode Statistics (HES) [21], General Practice Research Database (GPRD) [22], and routine KC-60 returns from STI clinics [23] (Table 4). The 3 sources identify cases from different care pathways, and there is an unknown degree of overlap between them. We assume that the total of the STI, GPRD, and HES data within each age group represents an upper bound for the number of PID cases diagnosed in England each year. A minimum was formed by adding the number of cases identified at genitourinary clinics to the largest of GPRD or HES cases [4]. A comparatively direct estimate of PID incidence can be derived from the control arm of the POPI trial [17] if we assume that the trial sample is approximately representative of the general female population of the same age, approximately 16–24 years. In the unscreened arm, 23 cases of clinical PID were reported in a sample of 1186 women aged 16–27 years followed up for 1 year.

**Table 4.** Number of Incident Cases of Diagnosed Pelvic Inflammatory Disease (PID) in England, 2002, by Data Source

Age	Hospital Episode Statistics	General Practice Research Database^a^	Genitorurinary Medicine Clinics^b^	Total Female Population
16–19 y	1233	5083	3212	1 199 600
20–24 y	3101	8842	4399	1 519 100
25–34 y	9756	14932	3919	3 502 100
35–44 y	10 526	9609	1388	3 795 600

^a^ Definite and probable PID, as defined by French et al [22].

^b^ Data by age not available for 2002, so we assumed that the age distribution for these data were the same as in 2009.

Routine data only represent diagnosed *“*probable/definite” PID. In the POPI trial, we assume that all symptomatic PID cases meeting the “probable/definite” criteria will be ascertained, including those normally undiagnosed [4, 20]. To account for this, we identified a single study providing estimates of the proportion of PID cases that are symptomatic and diagnosed [5]. This is a cross-sectional study of 36 women with TFI. Eleven reported a previous diagnosis of PID, 21 reported a history of symptoms but no diagnosis, and 4 reported no history of symptoms or diagnosis.

We used these data to generate 2 independent estimates of PID incidence in women. These estimates were found to be highly consistent [4], so all of the data were jointly synthesized to generate the pooled estimates shown in Table 3.

### Statistical Estimation

Estimation uses a Bayesian approach. Posterior medians and 95% CrIs were obtained from the Bayesian Markov chain Monte Carlo (MCMC) package WinBUGS, version 1.4.3 [24]. This method ensures that all of the uncertainty in the data is fully propagated into the estimates of PEF. Details of the prior distributions used are given in Appendix 2. To assess goodness of fit, we use the posterior mean residual deviance, whose expected value approximates the number of data points under the assumption that the model is true [25, 26]. Summary results are based on 2 chains with 200 000 samples each after a 50 000 burn-in period. Convergence was checked through visual inspection of trace and history plots and the Brooks-Gelman-Rubin statistic [27], which demonstrated convergence of all parameters within 1000 samples.

## RESULTS

**Figure 1.**
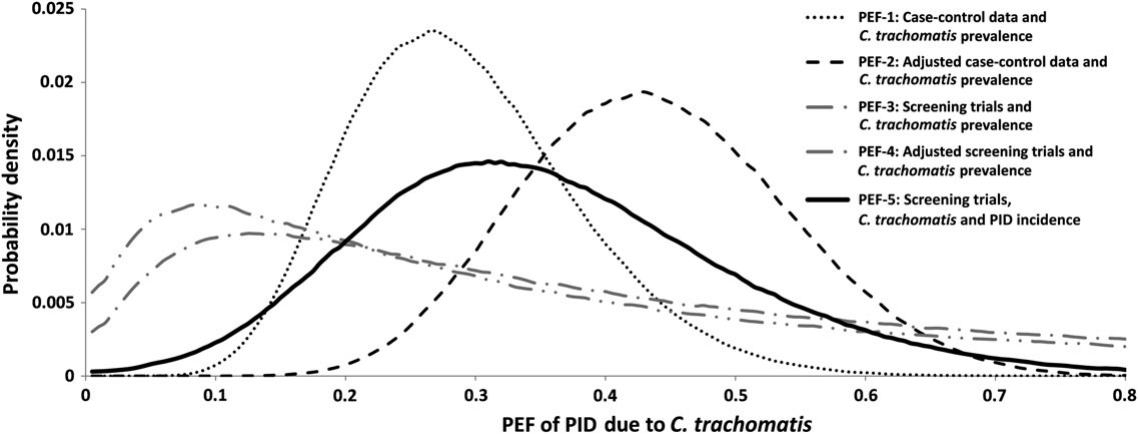
Evidence consistency plot showing the posterior densities for the 5 estimated population excess fractions (PEFs) in women aged 16–24 years. Posterior distributions are based on 950 000 samples in bins of size 0.005 with a 3-bin moving average smoother applied. See Appendix 2 for further details. Abbreviations: *C. trachomatis*, *Chlamydia trachomatis*; PID, pelvic inflammatory disease.

The 5 estimates of the PEF are shown in Table 5, and the full marginal posterior distributions for the age group 16–24 years are shown in Figure 1. Initial estimates of the PEF for women aged 16–44 years ranged from 12% to 20%, but following adjustment for biases, estimates ranged from 16% to 24%, showing a fair degree of consistency, although 95% CrIs were wide. Our preferred estimate, based on RCT evidence and national estimates of exposure and outcome, is 19.7% (95% CrI, 5.9%–38.1%).

**Table 5.** Alternative Estimates of the Population Excess Fraction (PEF) From Each Model

Age	${\text{PEF}}_{a}^{\left(1\right)}$ : Case-Control Data and *C. trachomatis* Prevalence^a^	${\text{PEF}}_{a}^{\left(2\right)}$: Adjusted Case-Control Data and *C. trachomatis* Prevalence^b^	${\text{PEF}}_{a}^{\left(3\right)}$: Screening Trials and *C. trachomatis* Prevalence^c^	${\text{PEF}}_{a}^{\left(4\right)}$: Adjusted Screening Trials and *C. trachomatis* Prevalence^d^	${\text{PEF}}_{a}^{\left(5\right)}$: Screening Trials, *C. trachomatis* and PID Incidence^e^
16–19 y	33.1 (16.9–54.8)	49.3 (29.4–70.3)	30.1 (1.1–93.4)	36.4 (3.1–94.8)	53.5 (15.6–100)
20–24 y	22.8 (11.1–41.4)	36.8 (20.4–58.0)	20.6 (0.7–89.5)	25.5 (1.9–92.6)	24.3 (7.2–47.6)
25–34 y	7.8 (3.4–17.2)	14.4 (6.7–28.8)	6.9 (0.2–71.1)	9.0 (0.6–76.0)	10.6 (2.9–21.2)
35–44 y	5.7 (2.3–13.5)	10.6 (4.6–23.3)	5.0 (0.1–63.7)	6.5 (0.4–69.2)	11.5 (3.0–25.7)
16–24 y	27.8 (14.0–47.9)	43.1 (25.0–64.2)	25.2 (0.9–91.6)	30.8 (2.5–93.4)	35.3 (10.5–68.5)
25–44 y	6.8 (2.9–15.1)	12.5 (5.8–25.8)	6.0 (0.2–67.7)	7.8 (0.5–72.9)	10.6 (3.0–21.9)
16–44 y	13.7 (6.4–27.1)	23.7 (12.3–42.0)	12.2 (0.4–81.8)	15.5 (1.0–85.3)	19.7 (5.9–38.1)

Data are posterior medians (95% credible intervals).

Abbreviations: *C. trachomatis, Chlamydia trachomatis*; PID, pelvic inflammatory disease.

^a^ Estimates are based on 3 case-control studies.

^b^ Estimates are based on 3 case-control studies, but the odds ratio was adjusted by data from the study by Erfurt et al [16] (see text).

^c^ Median PEF derived from the risk in the *C. trachomatis*–positive group relative to that in the *C. trachomatis*–positive and treated group in the POPI trial.

^d^ Median PEF derived from the risk in the *C. trachomatis*–positive group relative to that in the *C. trachomatis*–positive and treated group in the POPI trial, with adjustment for independent testing in the referral group.

^e^ Data are the ratio of the *C. trachomatis*–related PID incidence, estimated as the product of *C. trachomatis* incidence [11] and risk of progression from *C. trachomatis* infection to PID [20], to the all-cause PID incidence (Table 3) [4].

**Figure 2.**
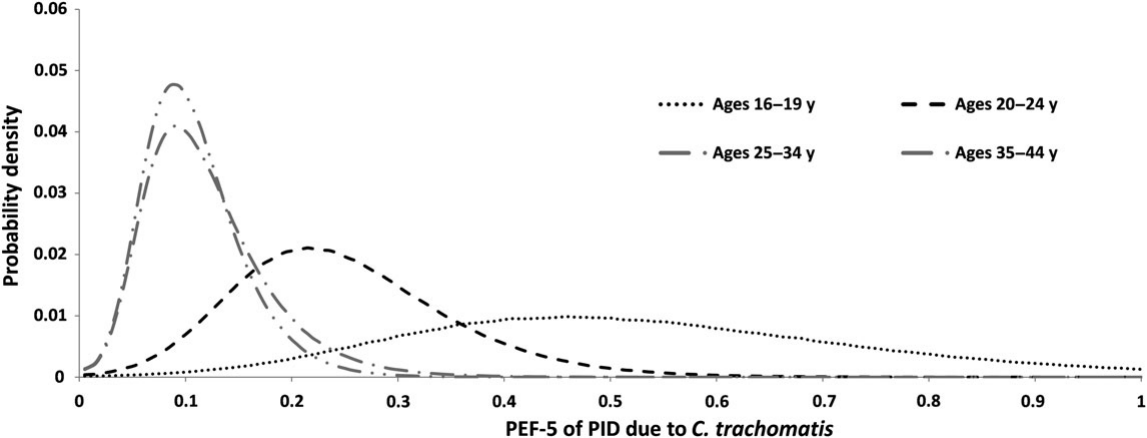
Posterior density plot for population excess fraction estimate 5 (PEF-5) by age group. Posterior distributions are based on 950 000 samples in bins of size 0.005 with a 3-bin moving average smoother applied. See Appendix 2 for further details. Abbreviations: *C. trachomatis*, *Chlamydia trachomatis*; PID, pelvic inflammatory disease.

In PEF-1 and PEF-2, the adjustment for underdetection of *C. trachomatis* in case-controls studies, based on the study by Erfurt et al, almost doubles the estimates of PEF within each age group (from 13.7% to 23.7% in women aged 16–44 years). The dramatic fall in PEF with age, from 49.3% in the group aged 16–19 years to 10.6% in the group aged 35–44 years, is a result of our assumption that the OR is not related to age, whereas the prevalence of *C. trachomatis* infection declines sharply (Table 2 and Figure 2).

PEF-3 and PEF-4 show a similar pattern with age. They are similar to PEF-1 but lower than PEF-2, owing to the estimated RR of 7.6 (95% CrI, 1.2–214.8) being lower than the estimated OR (especially the adjusted OR) from the case-control studies. However, the severe skew and very high variance of the posterior distributions reduce their value.

The PEF-5 estimate, based on the ratio of *C. trachomatis*–related PID to all-cause PID, is 19.7% (95% CrI, 5.9%–38.1%) in women aged 16–44 years and 35.3% (95% CrI, 10.5%–68.5%) in women aged 16–24 years and also shows a marked decline with age. This decline, from 53.5% to 11.5%, is a reflection of the assumption of a constant risk and the differing age profiles between *C. trachomatis* infection and PID incidence (Figure 2).

## DISCUSSION

We estimated the PEF of PID due to *C. trachomatis* in the United Kingdom in the period just before screening was introduced, using several different methods and data sources. We found reasonable consistency between the adjusted estimates. It is reassuring that adjusted estimates based on case-control studies are fairly close to estimates derived in a very different way from the progression risk from *C. trachomatis* to PID, *C. trachomatis* infection incidence, and all-cause PID incidence, although this is tempered by the high level of imprecision.

PEF drops by approximately 5-fold with age. In PEF-1–PEF-4, this is a consequence of applying an assumed constant OR to a prevalence that declines steeply with age. In PEF-5, the decline is due to the assumed constant risk, the decline in *C. trachomatis* infection incidence with age, the different age profile of PID in the routine data, and the assumption that the proportion of PID cases that are undiagnosed is constant over age. Age-dependency in PEF, if confirmed, has a significant impact on the public health importance of *C. trachomatis*, as the majority of ectopic pregnancies and TFI cases occur in older women. Although *C. trachomatis* infections in younger women may have a key role in reproductive health problems that emerge many years later, these results focus attention on the distinctly different age profiles of *C. trachomatis* and PID. If the risk profile changes with age, this may cause inconsistency in estimates of PEF between prospective and retrospective estimates. Studies following up women with *C. trachomatis* theoretically sample the (younger) *C. trachomatis–*infected population, whereas retrospective studies randomly sample the (older) PID population.

There are limitations to our analyses. The diagnosis of PID is imprecise, with no gold standard diagnostic test and changing criteria for diagnosing PID [2, 3, 6], with healthcare providers now being advised to maintain a low threshold for diagnosis [3]. Diagnosis of PID was usually obtained retrospectively, often by review of notes. Thus, there is also likely to be inconsistency in the use of and application of clinical criteria for the diagnosis of PID between the different studies. PEF-1 and PEF-2 use pooled ORs estimated from case-control studies. Owing to the likelihood of confounding, there is almost certainly an upward bias, as the risk factors for *C. trachomatis* infection are similar to the risk factors for other causes of PID, and the relevance of these studies to the United Kingdom is uncertain. PEF-3–PEF-5 are advantaged by being based on randomized comparisons reducing the risk of confounding. Finally, the decrease in these PEFs with age is in part due to our unavoidable assumption that neither the probability that PID is diagnosed nor the risk of PID following *C. trachomatis* infection, either directly in PEF-5 or through the assumptions of constant ORs and RRs in PEF-1–PEF-4, are age dependent. The rate of PID development can vary with age and past number of diagnosed *C. trachomatis* episodes [28]. But this does not inform whether risks from an individual (often undiagnosed) *C. trachomatis* infection vary. However, the degree of variation with age, by a factor of 4–6 between ages 16–19 years and age 35–44 years, is so great it would require extreme trends in one or both of those quantities to reverse it.

Our preferred estimate is PEF-5—19.7% (95% CrI, 5.9%–38.1%)—because it uses a synthesis of randomized evidence that accounted for clearance and reinfection to estimate risk in conjunction with population-level estimates of exposure and disease incidence. While there are good scientific reasons why we expect this to be the most reliable estimate, it is a post hoc decision.

Our estimates for the groups aged 16–44 years are lower than the estimate of 30% by Paavonen et al [7], mentioned previously from uncontrolled studies. Such studies likely overestimate the PEF as some of this exposure is likely coincidental. However, it has been argued [15] that this must also underestimate the current role of *C. trachomatis* infection, as gonorrhea was a common cause of PID during the period when many of these studies were undertaken, and it is generally agreed that gonorrhea is now far less common, particularly in Europe. Simms and Stephenson [8] found a range for the *C. trachomatis* infection prevalence of 12%–61% in the upper genital tract of women with laparoscopically proven PID, with considerable variation across countries. The largest United Kingdom study, conducted between 1989 and 1993, reported 39% [29]. In another United Kingdom study, conducted from 2000 to 2002, 42 of 140 salpingitis cases (30%) had evidence of exposure to *C. trachomatis*. We excluded these studies as they lack a control group, and none reported findings by age. However, if a control group had identified *C. trachomatis* in around 3% of women (roughly the prevalence in a population with the age distribution of PID cases), then an estimated PEF would be similar to our estimates. Such studies would typically require a similar adjustment to that described in PEF-2.

Our estimated adjustment factor from the study by Erfurt et al, 1.6, is close to an estimate by Taylor-Robinson et al, who observed that infection at the cervix appears to underestimate the role of *C. trachomatis* in PID-related reproductive damage by a similar amount [30]. This was based on the observation that, of the 22 women with acute salpingitis diagnosed on the basis of laparoscopy findings, 10 had *C. trachomatis* detected in cervical specimens, and an additional 6 had high-titer serum *C. trachomatis* immunoglobulin G antibody [30].

The focus of PID prevention strategies has centered on STIs, particularly *C. trachomatis* infection but also *Neisseria gonorrhoeae* infection and more recently *Mycoplasma genitalium* infection [3, 31, 32]. However, in the United Kingdom, gonorrhea is an uncommon cause [33], and an unknown but probably small proportion is caused by *M. genitalium* [3, 31, 32, 34]. If *C. trachomatis* is responsible for 20% of PID cases, then non-STI causes deserve more attention in PID prevention. These include microorganisms associated with bacterial vaginosis, which are commonly present in women with PID, and respiratory and enteric pathogens that have colonized the lower genital [3, 32, 35, 36]. Sexual exposure increases the risk of bacterial vaginosis [3, 35, 37], and sexual activity likely heightens the risk of ascending infection due to all pathogens. It is unknown whether the proportion of PID cases caused by non-STI microorganisms present in the vaginal microbiome [35] increases with age as the *C. trachomatis* PEF decreases, but this is possible [35, 36]. Currently, young age, multiple sex partners, and new partners, risk factors for STIs, are considered important risk factors when considering a diagnosis of PID in women who present with lower abdominal pain [3, 31, 38]. If older women are at increased risk of PID from non–STI-associated bacteria as the risk of *C. trachomatis* infection decreases, this needs to be recognized in clinical management guidelines for diagnosis and therapy [3, 31].

Future studies of the relationship between *C. trachomatis* and PID should focus more on the relationship between risk and age. Furthermore, age is a proxy measure for underlying factors such as cumulative incidence and immunological status. Serological studies using up-to-date assays in conjunction with other data sources may shed light on these more complex issues. Further work establishing the degree of overlap between cases identified in different routine data sources is required. Finally, more focus should be placed on investigation of the role of non-STI vaginal microbiota in PID.

## APPENDIX 1

### LITERATURE SEARCH**:** IDENTIFICATION OF CASE-CONTROL STUDIES OF *C. TRACHOMATIS* PREVALENCE IN PID CASES AND CONTROLS

We searched for studies using current *C. trachomatis* infection as a marker of *C. trachomatis* exposure. Strictly speaking, in a synthesis focusing on prevalence and sequelae of *C. trachomatis* in the United Kingdom, only contemporary United Kingdom data should be used. We have, nevertheless, used data from Europe because the epidemiology of STIs is generally similar in Western European countries. Studies from North America have been excluded because gonorrhea has tended to have a more important role in the etiology of PID in North America than in the United Kingdom [39–41]. We also excluded studies published before the 1990s. Our literature identification process is described in more detail elsewhere [4] and identified 3 studies shown in Table 1.

### META-ANALYSIS MODEL

The 6 data points are used to estimate 4 parameters: 3 study-specific “baselines”; the log odds in the control groups, *μ**_S_*, with *s* indexing study; and 1 fixed-effect log OR, *β.* Using a standard logistic regression model, with 0 for controls and 1 for PID cases,

$$\begin{array}{cc} & \mathrm{logit}(\lambda_{S,0})=\mu_{s}\\ & \mathrm{logit}(\lambda_{S,1})=\mu_{s}+\beta \end{array}$$

the OR can be recovered via the following equation: OR=exp⁡(β).

For PEF-2, data from the study by Hoyme et al [16] are used to place an informative prior on *ψ*, defined as the proportion of all cases of *C. trachomatis* infection in women with PID in which the organism is isolated from the lower genital tract, such that ψ∼Beta(56,47).

## APPENDIX 2

### PRIOR DISTRIBUTIONS

For the incidence rate and prevalence of chlamydial infection, a Multivariate Normal approximation (on the log and logit scales, respectively) to the full joint posterior distribution from the article by Price et al [14] is used. For PID incidence, the age-group specific rates of undiagnosed PID are given exponential(0.0001) priors. Exponential(0.01) priors give identical answers. The proportion of PIDs that are undiagnosed and the proportion of undiagnosed PIDs that are silent are given Beta(1,1) priors.

The OR in PEFs 1 and 2 is estimated using a logistic regression model with Normal(0,10 000) priors on the baseline and treatment parameters in the linear predictor. This is a fairly standard uninformative prior for this type of model. Assuming Normal(0,100) priors alters the (unadjusted) estimate from 9.2(4.4,18.1) to 9.1 (4.3,17.7). The adjustment factor from the study by Erfurt et al is also introduced as a Beta distribution derived directly from the data. The RR estimate for PEFs 3 and 4 is calculated as the simple ratio of the risks in each arm, which are given informative Beta prior distributions derived directly from the data. The adjustment factor for the POPI trial (used in PEF-4) is a Uniform distribution bounded between limits defined by the observed data. For PEF-5, the risk of PID due to a *C. trachomatis* infection is input as an informative Normal approximation to the posterior distribution estimated by Price et al [20].

### FIGURE-SMOOTHING ALGORITHM

Figure 1 shows density plots of the marginal posterior distributions for PEF-1–PEF-5. The 0-1 PEF proportion is split into n = 200 bins of size 0.005. For each PEF, the proportion of 950 000 raw MCMC draws falling into each bin y_n_ is recorded. *x* = 200 points are calculated using a 3 bin smoother such that $x_{1}=y_{1},\;x_{2}=\sum_{n=1}^{2}\;(y_{n}/2)$ , and $x_{n}=\sum_{n-2}^{n}\;(y_{n}/3)$ and for n = 3–200. The points x are used to plot the line graphs. The same smoother is used in Figure 2.

Figures 1 and 2 only show the posterior densities for the region 0 to 1. However, the PEFs are not bounded strictly within this region. PEF-1–PEF-4 are naturally bounded at 1 by the equations. This is not the case for PEF-5. Values of >1 were only sampled for PEF-5 in the 16–19-year age group, and these are not shown in the figure.

None of the PEFs are explicitly bounded at 0, and negative values were sampled for PEF-3 and PEF-4, as the distribution of the estimated RR does not rule out a protective effect from chlamydial infection. However, due to the extreme implausibility of this and difficulties with interpretation of negative PEFs calculated in this way, these are not displayed.

Note that, as we report posterior medians, the central estimates of PEF are unaffected by this. If upper or lower CrIs lie outside the range 0 to 1, these have been set to 0 or 1, respectively. Where necessary, the densities in Figures 1 and 2 have been rescaled slightly to integrate to 1 within the 0 and 1 boundaries.
